# Molecularly Imprinted Polymers for the Identification and Separation of Chiral Drugs and Biomolecules

**DOI:** 10.3390/polym8060216

**Published:** 2016-06-03

**Authors:** Sha Yang, Yonghui Wang, Yingda Jiang, Shuang Li, Wei Liu

**Affiliations:** Nano Structural Materials Center, School of Materials Science and Engineering, Nanjing University of Science and Technology, Nanjing 210094, Jiangsu, China; ys_njust@163.com (S.Y.); 13770729019@163.com (Y.W.); 613116002399@njust.edu.cn (Y.J.)

**Keywords:** molecularly imprinted polymer, chiral separation, chiral drugs, chromatography

## Abstract

Molecularly imprinting polymers (MIPs) have been extensively applied in chromatography for the separation of chiral drugs. In this review, we mainly summarize recent developments of various MIPs used as chiral stationary phases (CSPs) in high performance liquid chromatography (HPLC), capillary electrochromatography (CEC), and supercritical fluid chromatography (SFC). Among them, HPLC has the advantages of straightforward operation and high selectivity. However, the low separation efficiency, due to slow interaction kinetics and heavy peak broadening, is the main challenge for the application of MIPs in HPLC. On the other hand, CEC possesses both the high selectivity of HPLC and the high efficiency of capillary electrophoresis. In CEC, electroosmotic flow is formed across the entire column and reduces the heavy peak broadening observed in HPLC mode. SFC can modify the low interaction kinetics in HPLC when supercritical fluids are utilized as mobile phases. If SFC and MIP-based CSPs can be well combined, better separation performance can be achieved. Particles, monoliths and membrane are typical formats of MIPs. Traditional MIP particles produced by bulk polymerization have been replaced by MIP particles by surface imprinting technology, which are highly consistent in size and shape. Monolithic MIPs are prepared by *in situ* method in a column, greatly shortening the pre-preparation time. Some novel materials, such as magnetic nanoparticles, are integrated into the MIPs to enhance the controllability and efficiency of the polymerization. This review will be helpful to guide the preparation, development, and application of MIPs in chromatographic and electrophoretic enantioseparation.

## 1. Introduction

Most biomolecules and drugs exhibit chirality [1]. The separation of enantiomers for chiral molecules is crucial, particularly in the pharmaceutical industry, since enantiomers can present different, and even opposite pharmacological and toxicological properties [2]. A famous tragedy is the so-called “thalidomide disaster”: thousands of babies were born with malformed limbs due to misuse of *S*-thalidomide for pregnant women [3]. It was not until the 1980s that effective methods and techniques were developed to prepare drugs with highly enantiomerical purity [4,5,6,7,8]. Since then, high performance liquid chromatography (HPLC) has become the most extensively used approach for chiral separation, while capillary electrochromatography (CEC) is attracting increasing interest recently [9,10,11,12,13]. In enantiomer separation, chiral stationary phases (CSPs) largely affect the resolution and separation efficiency. A variety of materials can be employed as CSPs; typical examples include polysaccharides [14,15], proteins and DNA [16], cyclodextrins (CDs) [17,18,19,20], and molecularly imprinted polymers (MIPs) [21,22,23].

**Figure 1:**
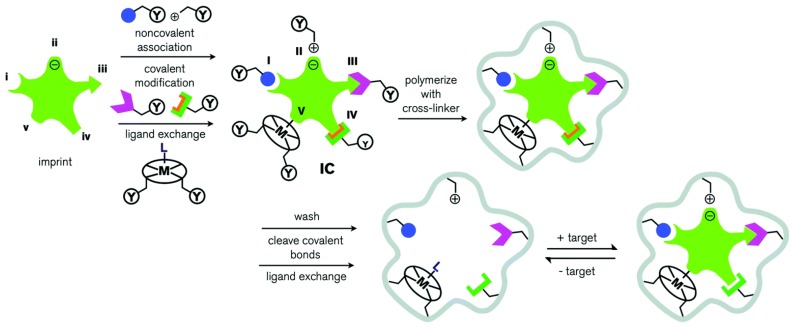
Schematic representation of the preparation, recognition, and separation of a molecularly imprinted polymer. There are five main types of molecular imprinting: (**i**) non-covalent; (**ii**) electrostatic/ionic; (**iii**) covalent; (**iv**) semicovalent; and (**v**) metal centre coordination. Template molecules and functional monomers, containing a functional group, Y, are combined by cross-linkers. After the polymerization progress, a three-dimensional polymer network is formed. Then, the template molecules are removed by washing, cleaving covalent bonds or ligand exchange, leaving cavities similar to template molecules in size, shape, and molecular interactions. By the addition of chiral compounds, the enantiomer similar to the template molecule will have a stronger interaction with the surrounding cavities. Reproduced with permission from [51]. Copyright 2014 The Royal Society of Chemistry.

MIPs have been exploited as CSPs since the pioneering work of Wulff in the 1970s. The main advantages of MIPs used as CSPs in chromatographic chiral separation are their high affinity and selectivity for the target molecule [21]. Furthermore, their production costs are relatively low. These advantages lead to the spread of applications for MIPs in many fields [24], such as separation science and purification [5,25,26,27], sensor technology [28,29,30,31], drug delivery [32,33,34], and artificial antibodies [35,36]. As shown in Figure 1, MIPs are produced based on a process in which template molecules and functional monomers are mixed into a complex. The functional monomer contains a functional group, Y, which undergoes a cross-linking reaction with an appropriate cross-linker. Then, a three-dimensional polymer network (grey) is formed, where template molecules are surrounded by monomers. After that, template molecules are removed, leaving cavities similar to template molecules in size, shape, and molecular interactions [21,37]. The creation of MIP is often classified into five categories: covalent, non-covalent, semi-covalent, electrostatic/ionic, and metal center coordination. The most widely applied technique is the non-covalent molecular imprinting method, which has many advantages, such as simple preparation, easy removal of template, and fast rebinding kinetics [38]. Methacrylic acid (MAA) is a commonly-used functional monomer in non-covalent imprinting, having excellent capability to interact with various functional groups like esters, acids, amides, and amine substituent.

Normal formats of MIPs for enantioseparation mainly include monoliths [39,40], particles [41,42,43], and membranes [44,45,46]. Traditionally, MIP-based CSPs were yielded by bulk polymerization [38]. These obtained bulk polymers should be crushed and ground. Such a format of MIP-CSP was extensively applied in chromatography due to its low cost and considerable physicochemical stability. However, grinding and sieving processes were difficult to realize subtle control. As a result, this procedure is extremely time-consuming and often leads to excessively irregular dimensions. Notably, most obtained CSPs are fit for the separation of small-to-medium sized molecules, but not for large molecules. Conversely, monolithic columns can efficiently separate large molecules like peptides and carbohydrates. Moreover, monolithic MIP stationary phases are simpler and cheaper than other types of MIPs [12]. Also, molecularly imprinted microspheres seem to be good candidates, which are usually synthesized by precipitation polymerization, showing highly uniform size and shape. However, this technique requires large volumes of the polymerization medium, which may hamper molecular interactions [47]. Finally, the polymerization progress is sensitive to changes in conditions, such as the identity and volume of solvent, formulation, template, and temperature, which would significantly influence the quality of MIPs [48]. When MIPs are employed as CSPs in practice, they may shrink or swell due to contact with mobile phases. The undesired deformation may break the three-dimensional network, rendering a failure for the use of chiral separation [49]. In this context, silica-based MIPs were generated, which present excellent mechanical strength and good solvent resistance [50,51].

In this review, we will introduce the developments and applications of MIP in HPLC, CEC, and supercritical fluid chromatography (SFC) in Section 2, Section 3 and Section 4 respectively. Different formats of MIPs as CSPs in HPLC and CEC are discussed in Section 2 and Section 3. Recent improvements in the preparation of MIPs, such as the introduction of molecular crowding, surface imprinting tecnique, and ionic liquid will be addressed in detail. We will also address the combination of MIPs with some advanced techniques (micro devices) and novel materials (nanotube, magnetic nanoparticles). In addition, Table 1 presents an overview of literatures involved in this review.

## 2. High Performance Liquid Chromatography (HPLC)

HPLC has been the most extensively applied technique for chiral separation in last several decades. HPLC was developed from liquid chromatography (LC) [52], and was firstly applied into business in 1969. In 1985, Mosbach’s group combined MIPs with the LC technique for the separation of amino acid derivatives [53]. Since then, MIPs have become increasingly popular as CSPs in HPLC.

### 2.1. Particles in HPLC

**Figure 2:**
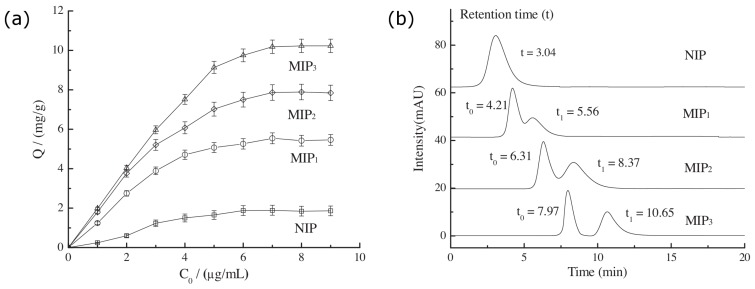
(**a**) Adsorption capacities of MIP${}_{1}$, MIP${}_{2}$, MIP${}_{3}$, and NIP; (**b**) Chromatograms of chiral separations of d-Phe and l-Phe with MIP${}_{1}$, MIP${}_{2}$, MIP${}_{3}$, and NIP as stationary phase, respectively. MIP${}_{1}$ used MAA as the functional monomer, MIP${}_{2}$ used acryloyl-β-cyclodextrin as the functional monomer, MIP${}_{3}$ used MAA and acryloyl-β-cyclodextrin as the binary functional monomer. NIP was employed without addition of imprinted polymer. The phenylalanine racemate solution concentration was 10 μg/mL. The mobile phase consisted of 85% acetonitrile (containing 1.0% HAc) and 15% water. MIP: molecularly imprinted polymer; NIP: non-imprinted polymer. Adapted with permission from [66]. Copyright 2012 Elsevier.

It is inefficient to prepare MIPs particles by bulk polymerization. These obtained particles also present poor separation behavior when they are used as CSPs. To solve this problem, the surface imprinting technique (SIT) emerged, which has been successfully used in the imprinted coating on a variety of nanomaterials [54]. Examples include the typical formats of silica particles [55,56,57,58], nanotubes [59], nanowires [60], and magnetic nanoparticles [61,62]. The SIT presents its significance for the formation of MIPs on the surface of support particles. The resultant MIPs show highly uniform size and shape. As a consequence, more efficient MIP particles can be obtained, leading to higher binding capacity, faster mass transfer, and easier adsorption and removal of templates than traditional MIP particles [63]. For example, Dong *et al.* [64] studied a CSP prepared by coating a MIP on the surface of silica gel (SMIP-CSP) for the separation of 2, 2${}^{\prime }$-diamine-1, 1${}^{\prime }$-binaphthalene (DABN) racemate. By means of the optimization of various parameters, including pretreatment temperature and the ratio between enantiomers and monomers, it shows a high separation factor (3.39) in HPLC mode, higher than their previous work (2.14) using MIPs produced by bulk polymerization [65]. The selection of functional monomers also has an extreme difference. A MIP l-phenylalanine (l-Phe) was synthesized based on monodisperse hybrid silica microspheres (MH-SiO${}_{2}$) with –CH=CH${}_{2}$ groups by surface imprinting technique [66]. β-cyclodextrins (CDs) were mixed into the functional monomers, which were sensitive to water soluble structures. The exterior of a β-CD is hydrophilic, while the cavity is hydrophobic. Separation properties of MIPs with different monomers were compared in Figure 2. MIP${}_{1}$ recognized l-Phe mainly by hydrogen-bonding (HB) interaction, while the adsorption capacity of MIP${}_{2}$ depended on hydrophobic effect. It was indicated that hydrophobic effect played a more important role than HB interaction. Importantly, MIPs with the binary functional monomers exhibited the best shape recognition toward l-Phe and d-Phe.

**Figure 3:**
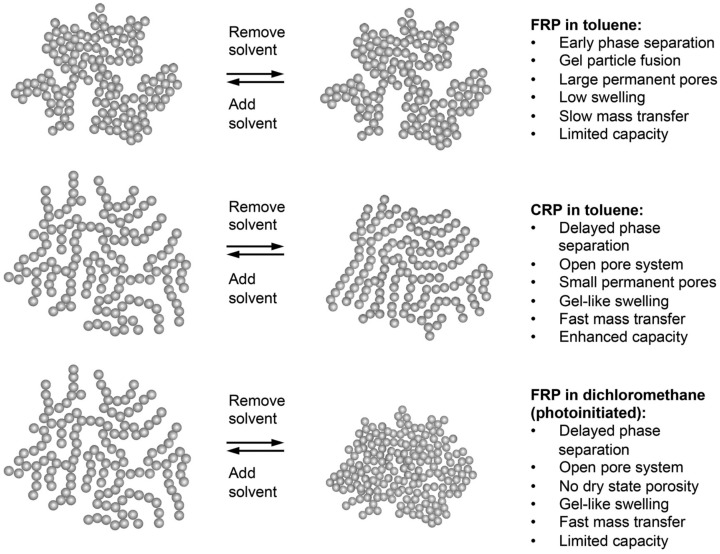
Pore systems deriving from different properties of imprinted network polymers prepared under different conditions. CRP: Controlled radical polymerization; FRP: Free radical polymerization. Reproduced with permission from [68]. Copyright 2015 The Royal Society of Chemistry.

The thickness of the thin film of MIP-based CSPs on the silicon-gel bead is a vital paramet=er that has a great influence on separation results. In fact, until now it remained a challenge to control the thickness and morphology. Raquel *et al.* [67] employed the iniferter-mediated grafting approach to develop a surface-imprinted chiral stationary phase for the separation of the enantiomers of the creitalopram. The ”grafting from” method was employed, in which the polymerization started from the surface if reactive groups were added beforehand. After MIP chiral selectors were grafted to the surface of porous silica particles, a homogeneous material was formed, which had a stronger interaction with the *S*-enantiomer of the drug. The ”grafting from” approach can effectively control the thickness of grafted polymer. There was an optimal thickness: higher or lower thicknesses would not provide the best resolution of the tested racemate. Controlled radical polymerization (CRP), especially addition fragmentation chain transfer polymerization (RAFT), presents its huge potential in tuning the morphology, complex framework, and functionality of a well-defined MIP [82,83]. RAFT polymerization is based on the addition of chain transfer agents (CTAs), such as dithioesters, which are particularly versatile [84]. RAFT has been proven to be able to reduce heterogeneity of resultant polymers. Mahadeo *et al.* [68] found that the addition of CTAs has a significant influence on the porous structure of network polymers (see Figure 3). Compared with free radical polymerization (FRP) in toluene, the yielded polymers through CRP in toluene had many superiorities, such as enhanced capacity, faster mass transfer, gel-like swelling, small permanent pores, and delayed phase separation, no matter whether it was in solvent condition or not. The RAFT would contribute much to the controllable development of MIP-based CSPs on the supporting materials to obtain excellent MIPs with high homogeneity and capacity.

### 2.2. Monolithic HPLC

Monolithic MIPs have recently been extensively applied in HPLC for chiral separation. Compared with particles, the preparation process of MIP monoliths are more straightforward and convenient. Matsui *et al.* [69] carried out a precursory research to synthesize MIP-based CSPs by means of the *in situ* method. In the MIPs preparation procedure, a template compound, a functional monomer, and a cross-linker were mixed in a stainless steel column and heated for polymerization. The polymerization occurred in the column, greatly shortening the pre-preparation time. Notably, the selection of the solvent is extremely important. Specifically, a suitable porogenic solvent should satisfy three criteria [70]: (1) Template molecules, initiator, monomer, and cross-linker must be soluble in the porogenic solvents; (2) The porogen should be able to create large pores, which can modify the flow-through property of the resulting polymer; (3) The porogenic solvents should have low polarity. Low polarity can have weak interferences to the interaction between the imprint molecule and the monomer during polymerization, being important to obtain MIPs with high selectivity. Based on these views, a mixture of toluene and dodecanol was employed as the porogenic solvent to fabricate molecularly imprinted monolithic stationary phases. The resulting monolith was applied for liquid chromatographic separation of enantiomers of amino acid derivatives. In this case, low back pressure can be obtained due to large through-pores, which would benefit the separation performance.

To improve the separation efficiency, more effective imprinted sites are needed. Commonly, the number of effective imprinted sites mostly depends on the ratio of monomers. However, the permeability is bad for traditional volatile organic solvents with high monomer content. Room-temperature ionic liquid (RTIL) attracts much attention due to its excellent properties, such as low vapor pressure, excellent solvation qualities, and good chemical and thermal stability [85]. Also, RTIL is a kind of green solvent, which has temperate effects on the environment. Significantly, an acceleration can also be observed when the polymerization proceeds in the ionic liquids [86]. Subsequently, RTILs present immense potential as replacements of traditional solvents in the MIP preparation process. For example, a MIP monolith was synthesized with good permeability in a solvent with ions [71]. The polymerization proceeded in a 1-butyl-3-methylimidazolium-tetrafluoroborate ([BMIM][BF4])-based solvent. A large amount of monomers were utilized in the mixture, beyond 80%. The yielded MIP monolith possessed high capacity and excellent permeability, and the process was found to be eco-friendly. Bai *et al.* [72] combined ionic liquid and the pivot strategy for chiral separation. Metal ions were used as pivots between the template and functional monomer, which strengthen the interactions between template molecules and functional monomers. The addition of metal ions into the pre-polymerization mixture led to smaller through-pores in the yielded monolith. The ILs in the porogen improved column efficiency and selectivity of metal ion-mediated MIP monoliths. Using metal ions as pivot during the MIP preparation process was proven to be an effective method to improve the separation performance of MIP, particularly suitable for the process occurring in a polar solvent.

### 2.3. Membrane in HPLC

Membrane is another MIP format. In the 1990s, MIP-based membranes were demonstrated to be feasible in HPLC for chiral separation [87]. New developments of MIP-based membranes have sprung up during recent years. Permselectivity and flux are important properties for membrane separation. In most cases, there is a trade-off relationship between the flux and permselectivity. Therefore, it is a challenge to improve the flux of a MIP membrane without deterioration of permselectivity. Sueyoshia *et al.* [73] synthesized two types of membranes: molecularly imprinted membranes (MIPMs) and molecularly imprinted nanofiber membranes (MINFMs). The flux from MINFMs is one-to-two orders larger than ordinary MIPMs. Meanwhile, the decline of permselectivity was not observed. Compared with traditional MIPMs, MINFMs can find more excellent potential in chiral separation. There are some other approaches to modify the properties of MIPMs, such as an appropriate selection of substrate. Qiu *et al*. [88] has prepared ractopamine MIPs nanotube membranes on anodic alumina oxide (AAO) nanopore surface by atom transfer radical polymerization. AAO has a highly-ordered hexagonal nanopore array as well as adjustable pore diameter, thickness and shape, so the resultant polymers usually have uniform shape and size. Moreover, AAO-MIPs have a small dimension with a high specific surface area. Therefore, most of template molecules are located at the surface or near the MIP surface. As a result, one can obtain a membrane with high binding capacity. The emergence of AAO as a nanoreactor for molecular imprinting can eliminate the drawbacks of traditional imprinting, such as incomplete removal of the template, small binding capacity, slow mass transfer, and irregularity in the shape of materials.

## 3. Capillary Electro Chromatography (CEC)

CEC is a mixed separation method that combines LC with capillary electrophoresis (CE). Such a hybrid technique possesses both the high separation efficiency of CE and the high selectivity of HPLC [89]. Although MIPs are extensively used in HPLC for chiral separation, they have some disadvantages, such as slow interaction kinetics and heterogeneity of binding sites [90]. Besides, peak broadening and tailing of the template molecule will be deteriorated when liquid is pumped through a column under an external pressure [11], leading to a parabolic flow profile in HPLC. In contrast, electroosmotic flow (EOF) in CEC is formed across the entire column. The flow is independent of the diameter of column, finally presenting an almost flat profile in the cross-section. Therefore, higher column efficiency can be achieved when separations are launched in CEC mode than in HPLC mode. In addition, CEC can minimize the consumption of samples. In view of the advantages of CEC method, many efforts have been made to apply MIP into CEC in recent years [12,90], mainly focusing on monolith, particles, and open tabular (OT) capillary columns.

### 3.1. Particles in CEC

Traditionally, to preserve imprinted sites of MIPs, a mass of cross-linkers should be utilized, which leads to an inflexible network. This results in the difficulty in the adsorption and removal of the template molecule. Recently, liquid crystalline polymer networks have been introduced to prepare MIPs [91]. In this case, imprinting sites can be preserved by the orientation deriving from the mesogenic side-groups. Notably, mesomorphic order can strengthen the interactions between the template molecules and the liquid crystalline-polymer network. Simultaneously, the shape memory of the imprinted cavities is reinforced. For example, Liu *et al.* [74] employed liquid crystalline monomers to synthesize low cross-linked MIP nanoparticles. The yielded MIP nanoparticles were applied in CEC to separate enantiomers of racemic zopiclone, presenting qualified peak symmetry. Their results indicated that the liquid crystalline MIP nanoparticles were more homogeneous and showed higher reproducibility of CEC than traditional MIP nanoparticles with high-level cross-linker.

Nanoparticle has a great influence on the performance of chiral separation. Liu *et al.* [75] prepared *d*-zopiclone-imprinted nanoparticles by precipitation polymerization and obtained MIP particles with sizes below 80 nm. The polymerization process runs in the diluted pre-polymerization mixtures. A base-line resolution of two enantiomers was achieved in CEC mode, and no obvious tailing peak was observed. Under the condition of particles with small enough size, the ratio of cross-linker to functional monomer and template to functional monomer had no influence on the performance of chiral separation and efficiency of columns with MIP nanoparticles.

**Figure 4:**
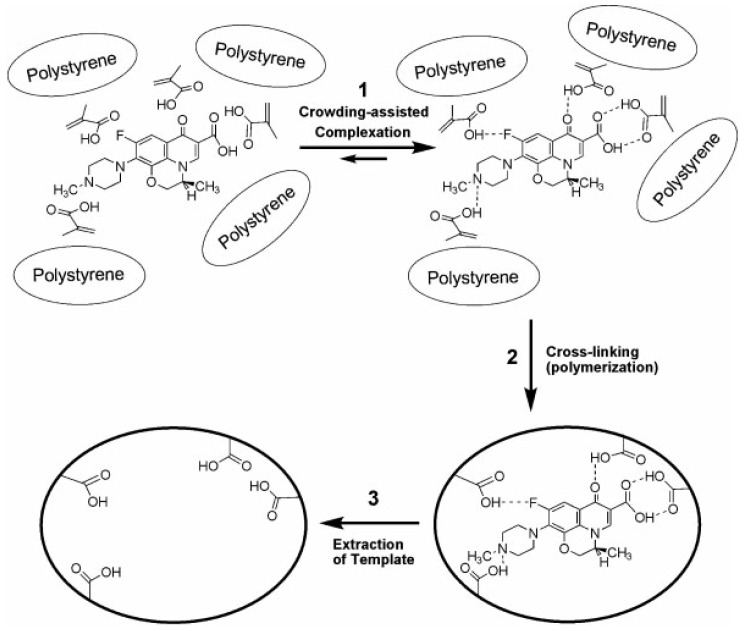
Schematic representation of molecular imprinting under molecular crowding conditions. *S*-ofloxacin (a template molecule) was mixed with methacrylic acid in tetrahydrofuran with polystyrene as a macromolecular co-solute. Then *S*-ofloxacin was cross-linked with ethylene glycol dimethacrylate by heating. After the polymerization, template was removed from the matrix and left a binding site. Reproduced with permission from [76]. Copyright 2011 Wiley.

Recently, a new strategy named crowding was generated to synthesize MIPs with high capacity [92]. In the case of molecular imprinting, molecular crowding can intensify the interaction between molecules and facilitate framework formation of template molecules and functional monomers. The molecular crowding strategy has been employed in the preparation process of MIP nanoparticles [76]. As shown in Figure 4, polystyrene-tetrahydrofuran (PS-THF) was employed as the crowding agent. The induction of PS-THF promoted the formation of available pores. Compared with traditional MIP prepared with acetonitrile (ACN) or chloroform, the resulting MIP based on a molecular crowding strategy showed higher selectivity and efficiency.

### 3.2. Open Tabular and Monolithic CEC

Among various formats of MIPs in CEC, the highest separation efficiency and resolution have been obtained in MIP coatings for OT CEC [90]. However, OT column with MIP coatings usually has low capacity. Recently, many efforts have been addressed to obtain MIP coatings with homogeneous binding site and large capacity. It was found that the imprinted coating columns at low levels of cross-linking showed faster separation, higher column efficiency, and more stable CEC results compared with that at high levels of cross-linking [91].

One-monomer molecularly imprinted polymers have recently been developed, which combine the functional binding to template with the corresponding cross-linking features for molecular recognition and polymer network formation. This methodology avoids tuning variable parameters, such as selection of functional monomer and crosslinker, the ratio of functional monomer to crosslinker, and the ratio of functional monomer to template. As a result, the design of a well-defined MIP is highly simplified. For example, using 2-methacrylamidopropyl methacrylate (MAM) as single functional monomer, a MIP coating was synthesized in fused silica capillary columns [77]. As a result, binding and selectivity performances were improved. The resulting MIPs were employed for the chiral separation of racemic amlodipine, naproxen, and ketoprofen, and a high resolution (16.1) of enantiomers separation was achieved. Zhao *et al.* [78] prepare POSS (polyhedral oligomeric silsesquioxanes)-based MIP by means of one-monomer strategy and employed it for the separation of enantiomers of naproxen, amlodipine, and andzopiclone in CEC mode. Combination of the specific features of a POSS network with the architecture of a MIP coating leads to higher separation efficiency, compared with that under conditions using MIPs without the presence of POSS. Under optimal conditions, the resolution was up to the high value of 22.3.

Monolithic columns still present its excellent potential in CEC. Its various merits have made monolithic columns attractive alternatives to commonly-used packed and open-tubular columns. Zong *et al.* [79] prepared a molecular crowding-based MIP monolith for CEC. The yielded MIP monolith showed better column permeability and higher chiral separation ability than MIP monolith without molecular crowding. Liao *et al.* [80] employed pressure-assisted CEC for the separation of polar nitroimidazole drugs. Polymethacrylate-based MIP monolithic column was synthesized, application of pressure by using a HPLC pump was involved during the polymerization process. The additional pressure greatly shortened the analysis time. Additionally, the pressure-assisted CEC exhibited excellent reproducibility.

### 3.3. Microchip Electrochromatography

**Figure 5:**
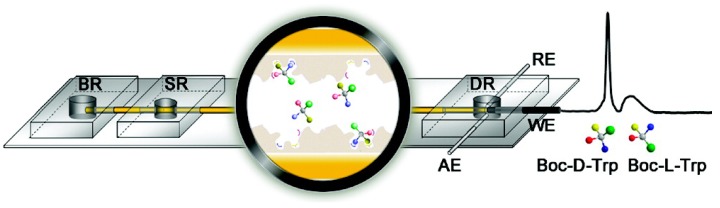
Schematic representation of the enantioseparation of chiral compounds on a MIP-coated microchannel of a microfluidic device. The MIP-coated microchannel in the microfluidic device was first washed by methanol/acetic acid (9:1, *v/v*), which was filled in the buffer reservoir (BR) and detection reservoir (DR). The fracture sampling was performed under a voltage of 200 V between the sample reservoir (SR) and BR. The corresponding separation voltage was applied to the BR with the DR grounded and the SR floating by automatically switching the high-voltage contacts. Finally, the electropherogram was recorded on a electrochemical station, using the “amperometric i–t curve” mode at an applied potential of +1.2 V. WE, working electrode; RE, reference electrode; AE, auxiliary electrode. Reproduced with permission from [93]. Copyright 2009 American Chemical Society.

**Figure 6:**
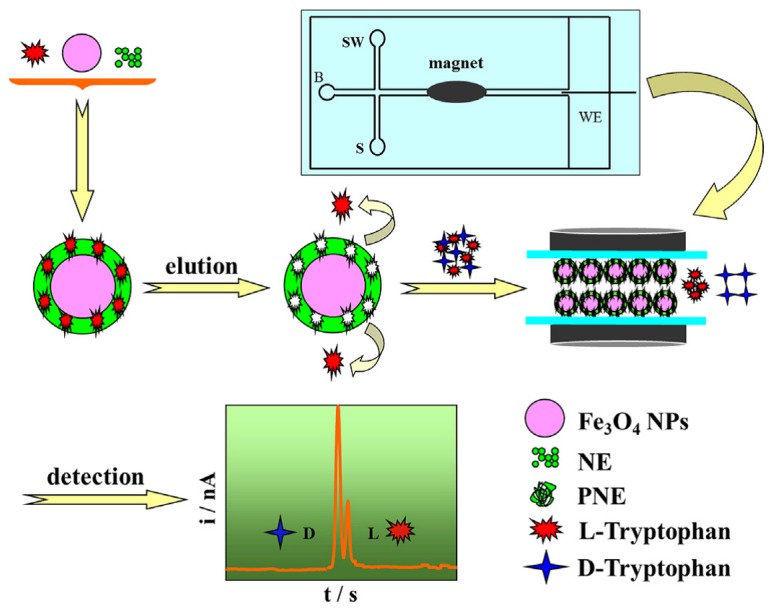
Schematic illustration of MIP-Fe${}_{3}$O${}_{4}$@PNE nanoparticles preparation and the enantioseparation on MIP-Fe${}_{3}$O${}_{4}$@PNE nanoparticles-packed microchannel. Firstly, the MIP-Fe${}_{3}$O${}_{4}$@PNE nanoparticles were agitated by autopipette mixing to ensure a consistent suspension. Then, the MIP-Fe${}_{3}$O${}_{4}$@PNE nanoparticles were packed into the microchannel by vacuum. The channel was filled with MIP-Fe${}_{3}$O${}_{4}$@PNE nanoparticles after continuous injection for about 5 min. During the separation process of d-tryptophan and l-tryptophan, the imprinted cavities presented a stronger interaction with l-tryptophan. PNE: polynorepinephrine. Reproduced with permission from [94]. Copyright 2015 Elsevier.

Recently, it has become a trend to combine MIPs with microchip electrochromatography (MCEC) for the faster enantioseparation of chiral drugs and biomolecules. Qu *et al.* [93] applied MIP into microchip electrophoresis (MCE) for fast enantioseparation of model enantiomers, tert-butoxycarbonyl-d-tryptophan (Boc-d-Trp) and Boc-d-Trp (see Figure 5). The MIP preparation was conducted on the inner wall of a microchannel by *in situ* polymerization. Acrylamide (AM) and ethylene glycol dimethacrylate (EDMA) were used as a functional monomer and cross-linker, respectively. The highly porous polymer film linked to the inner wall provided efficient interaction between the target molecules and the MIP. Molecular recognition to Boc-Trp enantiomers was proven to be efficient in the MIP-coated microchannel of microfluidic device (MIP-MCMD), presenting a baseline enantioseparation. Importantly, the analysis procedure was found to be reproducible and stable. Compared with conventional molecular imprinting techniques, the advanced method showed highly shortened analytical time, and less reagent and sample consumption. Subsequently, a microporous monolithic imprinted microchannel was fabricated for fast and sensitive chip-based enantioseparation [81]. Plentiful through pores were observed in the inner wall of imprinted microchannel. The resulting MIP monoliths presented excellent properties, such as large specific surface area, outstanding permeability, high flow rate, and efficiency. The imprinted capillaries were fixed in a poly dimethyl siloxane (PDMS) substrate on a glass slide. The rapid enantioseparation of racemic Tyr was realized with good resolution and high efficiency. Chen *et al.* [94] prepared a MIP-Fe${}_{3}$O${}_{4}$@polynorepinephrine (PNE) nanoparticles stationary phase-based microchip electrophoresis system (see Figure 6). The addition of Fe${}_{3}$O${}_{4}$ into the MIP leads to easy collection and isolation by an external magnetic field, avoiding additional centrifugation or filtration processes. The resultant MIP-Fe${}_{3}$O${}_{4}$@PNE presented an excellent enantioseparation property by combining the strong magnetism and high specific area of MIP-Fe${}_{3}$O${}_{4}$ with versatile groups and good biocompatibility of PNE. This microchip electrophoresis can be used to separate other kinds of chiral compounds by simply altering types of template molecules. On-chip separation of multiple chiral compounds can run simultaneously by simply mixing the MIP-Fe${}_{3}$O${}_{4}$@PNE nanoparticles-based stationary phase with relative template molecules.

## 4. Supercritical Fluid Chromatography (SFC)

Recently, SFC has attracted increasing interest in the chiral separation of drugs due to its advantages, such as higher efficiency and consuming less time than HPLC [95]. These advantages arise from the characteristics of supercritical fluids (SCFs). SCFs are formed under the condition that temperature and pressure of a gas or liquid exceed their critical values. SCFs have unique features, falling in between gas and liquid state. SCFs are suitable as major mobile phase components in chromatography due to many advantages, such as large diffusivity, low costs, and non-pollution [96]. Additionally, they can reduce the consumption of toxic solvents and additives. As such, it is attractive to combine the high selectivity of MIPs with the high efficiency of SFC [25].

Although SFC has many merits, few studies focus on the application of MIP-based CSPs in SFC during recent years. Ellwanger *et al.* [97] employed MIPs in SFC for chiral separation. Although MIPs were proven to be feasible as CSPs in SFC, unsatisfactory separation results were obtained. Slow interactions at the sites still existed, leading to a inefficient mass transfer under the SFC mode. However, MIPs still showed a potential as CSPs in SFC for enantioseparation. They put forward some expectation that the used MIP should have the properties of highest affinity, long-term stability, and a narrow site distribution. In 2012, Ansell *et al.* [98] employed MIPs as stationary phases in SFC. Notably, no depression of separation performance was observed, compared with HPLC condition. The method was reproducible and was confirmed in 21 different (−)-ephedrine imprinted polymers for the enantioseparation of (±)-ephedrine. The results were at least as good as that when MIPs were applied as stationary phases in HPLC. Their experiments further validated that MIP can find its potential for use as CSPs in SFC. Although no highly outstanding improvement was obtained, the potential of combination between MIPs with SFCs should not be overlooked.

## 5. Conclusions

Numerous studies have demonstrated the excellent performance of MIP in chromatographic enantioseparation. The introduction of the surface imprinted technique allows MIP particles to present highly uniform size and shape, improved selectivity, and higher efficiency, compared with traditional particles made by bulk polymerization. Some materials with unique properties—such as magnetic nanoparticles—are employed in the MIP preparation process. The presence of magnetic nanoparticles leads to novel MIPs, which can be easily controlled and isolated in an external magnetic field.

Monolithic MIPs prepared by *in situ* polymerization often possess low separation efficiency and selectivity. Some strategies, such as the molecular crowding method and metal ions as pivots, have been utilized to improve the separation performance. Besides, MIP monoliths can be integrated with micro devices to obtain highly-efficient chiral separation. To improve the flux of MIP membranes, a crucial parameter for separation, nano fibers have attracted much attention, due to their larger flux and binding capacity than traditional MIP membranes. The choice of substrate also makes a critical difference.

A better control of the morphology and framework of particles is in high demand to obtain a well-defined MIP with great homogeneity and high capacity. Although the highest resolution can be achieved in open tabular CEC, low capacity limits the application of MIPs in chiral separation. Further efforts will be contributed to obtaining a MIP-based enantioseparation with higher selectivity, efficiency, and reproducibility through some novel techniques—such as micro device, controllable methodology, and special materials like nanofibers and magnetic particles.

## Figures and Tables

**Table 1:** Overview of recent chiral separations based on MIPs. The resolution was calculated by 2(*t*${}_{2}$-*t*${}_{1}$)/(*w*${}_{1}$-*w*${}_{2}$), where *t*${}_{2}$ and *t*${}_{1}$ denote the retention time of two enantiomers, and *w*${}_{1}$ and *w*${}_{2}$ indicate the baseline of two enantiomers. 4-VP: 4-vinylpyridine; ACN: Acetonitrile; β-CD: β-cyclodexrin; [BMIM][BF4]: 1-butyl-3-methylimidazolium- tetrafluoroborate; CEC: Capillary electrochromatography; DMAEMA: Dimethylaminoethyl methacrylate; DMF: dimethylformamide; DMSO: dimethyl sulfoxide; EDMA: ethylene glycol dimethacrylate; EGDMA: ethylene glycol dimethacrylate; HEMA: 2-hydroxyethyl methacrylate; MCEC: Microchip electrochromatography; MAA: Methacrylic acid; MAM: 2-methacrylamidopropyl methacrylate; OT: Open tabular; PMMA: poly (methyl methacrylate); PS: Polystyrene; THF: tetrahydrofuran; TRIM: trimethylolpropane trimethacrylate.

Mode	Format	Template	Functional monomer	Cross-linker	Mobile phase/electrolyte	Solvent	Resolution	References
HPLC	Particle	*R*-BNA	MAA	EGDMA	ACN	-	1.25	[64]
	Particle	l-phenylalanine	MAA/acryloyl-β-CD	EGDMA	85% ACN (containing 1.0% HAc), 15% water	ACN/water (17:3, *v/v*)	1.46	[66]
	Particle	*S*-citalopram	itaconic acid	EDMA	formate buffer (40 mM, pH 3)/ACN (30:70, *v/v*)	ACN	1.80	[67]
	Particle	l-phenylalanine anilide	MAA	EGDMA	MeCN/sodium acetate buffer (10 mM, pH 4.8) (9:1, *v/v*)	MeOH/H${}_{2}$O (80:20, *v/v*)	1.53	[68]
	Monolith	l-phenylalanine anilide, d-phenylalanine anilide	acrylic acid	EDMA	-	cyclohexanol, 1-dodecanol		[69]
	Monolith	*N*-(carbobenzyloxy)-l-tryptophan, Fmoc-l-tryptophan	MAA, 4-VP	EDMA	ACN/acetate buffer (10 mM, pH 3.5) (40:60, *v/v*)	toluene/dodecanol	1.67	[70]
	Monolith	ketoprofen	4-VP	EDMA	ACN/acetate buffer (50 mM, pH 3.6) (99/1, *v/v*)	[BMIM][BF4]/DMSO (4:1, *v/v*)		[71]
	Monolith	*R*-mandelic acid	4-VP	EDMA	ACN/NaAc-HAc buffer (9:1, *v/v*) (50 mM, pH 3.6)	DMSO/DMF /[BMIM]BF4/Co${}^{2+}$	1.87	[72]
	Membrane	ractopamine	MAA	EGDMA	phosphoric acid buffer/ACN (0.1:99.9, *v/v*)	Dimethyl sulfoxide		[73]
CEC	Particle	*d*-zopiclone	MAA/liquid crystal	EDMA	ACN/acetate (20 mM, pH 3.6) (80/20, *v/v*)	toluene/ACN (7:3, *v/v*)	3.29	[74]
	Particle	*d*-zopiclone	MAA	EDMA	ACN/acetate-sodium acetate buffer (20 mM, pH 3.6) (85:15, *v/v*)	ACN	4.75	[75]
	Particle	*S*-ofloxacin	MAA	EDMA	ACN/acetate-sodium acetate buffer (10 mM, pH 5.0) (90:10, *v/v*)	polystyrene(crowding agent) -tetrahydrofuran	1.53	[76]
	OT	*S*-naproxen, *S*-ketoprofen, *S*-amlodipine	MAM	MAM	ACN/acetate (0.01 mM) (80/20, *v/v*)	toluene/isooctane (7:3, *v/v*)	16.10	[77]
	OT	*d*-zopiclone	MAA	EDMA, MAM, TRIM	ACN/acetate (0.05 mM, pH4.2) (80:20, *v/v*)	Toluene/isooctane (9:1, *v/v*)	22.30	[78]
	Monolith	*d*-zopiclone	MAA	EDMA	ACN/acetate (20 mM, pH 5.0) (85:15, *v/v*)	PMMA-THF, PS-THF	2.09	[79]
	Monolith	*S*-ornidazole	HEMA/DMAEMA (1:1, *v/v*)	EDMA	ACN/sodium dihydroge-nphosphate- phosphoric acid buffer (5 mM) (30:70, *v/v*)	Toluene/dodecanol	7.76	[80]
	MCEC	l-tyrosine	AM	EDMA	ACN/acetate buffer (50 mM, pH 4.0) (90:10, *v/v*)	ACN/isooctane (2:1, *v/v*)	2.40	[81]

## Abbreviations

4-VP: 4-vinylpyridine; AAO, Anodic alumina oxide; ACN, Acetonitrile; AM, Acrylamide; [BMIM]BF${}_{4}$, 1-butyl-3-methylimidazolium-tetrafluoroborate; CD, Cyclodextrin; CE, Capillary electrophoresis; CEC, Capillary electrochromatography; CRP, Controlled radical polymerization; CSP, Chiral stationary phase; CTA, Chain transfer agent; DABN, 2,2${}^{\prime }$-diamine-1,1${}^{\prime }$-binaphthalene; DMAEMA, Dimethylaminoethyl methacrylate; DMF, dimethylformamide; DMSO, dimethyl sulfoxide; EOF, electroosmotic flow; EDMA, Ethylene dimethacrylate; EGDMA, ethylene glycol dimethacrylate; FRP, free radical polymerization; HPLC, high performance liquid chromatography; HEMA, 2-hydroxyethyl methacrylate; LC, liquid chromatography; MAA, methacrylic acid; MAM, 2-methacrylamidopropyl methacrylate; MCEC, microchip electrochromatography; MINFM, microchip electrochromatography; MIP, molecularly imprinting polymer; OT, open tabular; PS, polystyrene; POSS, polyhedral oligomeric silsesquioxanes; PDMS, polyhedral oligomeric silsesquioxanes; PMMA, poly (methyl methacrylate); PNE, polynorepinephrine; SFC, supercritical fluid chromatography; SCF, supercritical fluid; SIT, surface imprinting technique; THF, tetrahydrofuran; TRIM: trimethylolpropane trimethacrylate.
